# Exploring and Validating the Effects of Mega Projects on Infrastructure Development Influencing Sustainable Environment and Project Management

**DOI:** 10.3389/fpsyg.2021.663199

**Published:** 2021-04-16

**Authors:** Tao Xiaolong, Nida Gull, Shahid Iqbal, Muhammad Asghar, Ahsan Nawaz, Gadah Albasher, Javaria Hameed, Ahsen Maqsoom

**Affiliations:** ^1^School of Business Administration and Tourism Management, Yunnan University, Kunming, China; ^2^School of Management Sciences and Engineering, Yanshan University, Qinhuangdao, China; ^3^Management Studies Department, Bahria University, Islamabad, Pakistan; ^4^Collage of Civil Engineering and Architecture, Institute of Construction Project Management, Zhejiang University, Hangzhou, China; ^5^Department of Zoology, College of Science, King Saud University, Riyadh, Saudi Arabia; ^6^Asia-Australia Business College, Liaoning University, Shenyang, China; ^7^Civil Engineering Department, COMSATS University Islamabad, Islamabad, Pakistan

**Keywords:** project management, environmental protection, economy boosting, project success, CPEC, sustainable development

## Abstract

The study is based on validating and exploring the effects of a mega project plan (CPEC) on infrastructure development and Sustainable Project Management. The CPEC has great importance to infrastructure development and economy-boosting. The current study's primary aim is to deal with environmental protection, economic boost up, international relations influencing to the Project's success. The paper also addressed project management as a moderator between environmental protection, economic boost up, international relations, and the CPEC project's success. The primary data has been gathered by using questionnaires, and PLS-SEM has been employed for the analysis. The results revealed that environmental protection, economy boost up, and international relations have a positive association with the success of CPEC. The outcomes also exposed that project management moderating among the nexus of economy boosts up the international relations and success of CPEC. The present study results guided how Pakistan and China make the CPEC project stronger with the efficient implementation of practices required for protecting the environment, with the economic growth and boost up, and good strong relations with foreign countries. This study was an attempt to validate the different factors to check their association with each other in a new environment, resulting in a leading edge for the success of mega projects that influence project management.

## Introduction

The CPEC is the initiative of China's one belt one road initiative. The development of infrastructure and other improvements in economic, political, environmental, and regional aspects is tremendous. However, there are a lot of challenges and concerns in the development and establishment of this project. Security and environment protection are two critical areas to be considered in this regard. Under this CPEC program, several modern and groundbreaking initiatives have begun. The building of the rail network, new economic zones, Gwadar port development, and road network initiatives are part of this significant economic development (Kanwal et al., 2020). Economic zones and other such infrastructure ventures need efficient transport. The decline in corruption would boost overall economic growth as far as sustainable growth is concerned (Abdullah et al., 2021). The loans for capital growth amounted to $11 billion. These loans certainly help Pakistan's economic growth. Another significant industrial growth development is that a building project on liquefied oil and gas pipeline has already started (Shahbaz et al., 2019; Mamirkulova et al., 2020; Sarfraz et al., 2020a,b; Ullah et al., 2020; Zaman et al., 2021).

Many new and innovative projects have been started under this CPEC initiative. Railways network construction, new economic zones, Gwadar port development, and road network construction initiatives are part of this substantial economic development (Li et al., 2020). Economic zones and other such projects development need efficient transportation. The road network optimization and new highway construction are also included in this project's developmental goals (Liu et al., 2016). The new and emerging trends of the CPEC also supported the economy of both countries. It is observed that foreign investors' trading investments have increased tremendously after the announcement and initiation of CPEC between Pakistan and China (Sial, 2014; Carvalho and Rabechini Jr, 2017; Wagner and Tripathi, 2018; Kanwal et al., 2019).

The economic and energy generation sectors are surely improved after initiation of this project, but the thing that should be considered is to avoid all the environmental losses (Khwaja et al., 2018; Initiative, 2020). Environment risk assessment should be considered as a prime responsibility of the Environment Protection legislation of both countries. Pakistan and China both are densely populated countries, and environmental pollution is a grave hazard in the way of CPEC project management (Hao et al., 2020a). Air quality and water resource scarcity are two major concerns. Water and air quality are adversely affected by the construction of roads. Biodiversity and natural reservoirs are also exhausted. These things should be considered effective, and management practices must be improved to support all these projects' development (Sarfraz et al., 2018a, 2020c; Nawaz et al., 2019, 2020; Huo et al., 2021). The international cooperation and support from other countries of the world are also the fruits of CPEC initiative. In the year 2018, Saudi Arabia has entered this project as a third partner and invested $10 billion. This investment will surely improve the pace of developmental activities. Saudi Arabia is investing in developmental projects and is also interested in the development of the mining industry of Pakistan (Jia et al., 2021). So, International relations are truly improved by this project. The development of infrastructure has significantly helped shape the economies and the associated development dimensions of both countries. Professional employees' exchange has generated several new programmers for social growth (Nawaz et al., 2021). The latest articles also addressed the management of energy and green innovation and scientific implications (Han et al., 2019). The exchange of social and economic wealth has clearly influenced the overall economic situation of entire Asia. Otherworld economies have invested in similar CPEC ventures too (Li et al., 2021), so it is safe to conclude that CPEC is hope and promotion to the Pakistani market and a conduit for economic growth and worldwide prosperity (Chakma, 2019; Kanwal et al., 2019).

The increased number of infrastructure development efforts and an influx of a huge number of tourists in the exotic natural reservoirs and natural parks of Pakistan has posed a grave environmental hazard to the tourist's spots and the biodiversity of those areas (Lin et al., 2015; Zeng et al., 2015; Winter, 2016). The combined efforts to support the rehabilitation and protection of these areas are necessary. The research evidence has provided deep insight into all the hazards which the environment has faced. Recently, a study conducted in Gilgit Baltistan showed that the climate change and air quality index of this mountain-based tourist spot has adversely affected construction procedures and the influx of tourists in these areas. The geographical location of Gilgit Baltistan is unique and is the central spot in CPEC (Khan et al., 2021). Air quality improvement and water resource management plans must be implemented in this area (Wu et al., 2020; Huo et al., 2021).

The environmental protection role is essential for any business or project's success (Sadiq et al., 2020a,b). Similarly, it is taking an essential role in CPEC Success. In a comfortable and suitable environment, any business will grow in a concise time. CEPC is the abbreviation of “China Pakistan Economic Corridor.” CPEC, officially launched in 2015 and completed in 2030 (Hao et al., 2020b). Under environmental protection, it would be very easy for anyone to work and perform better. And the company or project will grow accordingly. CPEC is a hub, connecting the Middle East, Europe and Africa with China and will generate a lot of economic activity for Pakistan. Roads are very important for this region. If the environment is good, then the people will prefer to live in such areas, which is very important for CPEC success (Chen et al., 2017).

The economy of any country is mainly dependent upon trading and industrialization. CPEC and its related projects provided a lot of ease in both sectors. It can be easily illustrated by the recent studies conducted by different researchers that the unemployment rate is greatly reduced due to enhanced investments and the development of new projects. The foreign investments have elevated the stocks and trade industry of both countries. The economic security and advancement depend upon the combined efforts of individuals of these two neighboring countries. The easy access to trading centers of China improved trading in Pakistan. Local traders and industrial heads have declared the CPEC as the key to a new era of opportunities (Maqsoom et al., 2021).

Economy boost-up will be due to CPEC because it will provide the opportunities. Companies from all over the world will take an interest to start bunnies here. It will also provide job opportunities which would be the one of the reasons for economic boosting. A competitive environment will be created due to this Market. Therefore, the best quality products will be available due to this competition. It would be a central point between the Gulf, Europe, and Asia (Ali et al., 2021). This network will stretch from China to the Arabian Sea in three parallel sections along eastern. CPEC can offer infinite bounties to many countries that can benefit the long run (Wu et al., 2021). These corridors will build an international market. Through this market, the trade will be easy and inaccessible to worldwide (Hao et al., 2020b).

The skill development in terms of the digitalized system is necessary, and Pakistan's government is working hard in this area. IT and artificial intelligence-related programs are made necessary to be included in all universities courses (Hao et al., 2020a; Nawaz et al., 2021).

Project management and related planning are essential in mediating the success factor of CPEC. Environmental protection, Economy boost-up, and International relations are all parts of the project management-related aspects (Chaudhri, 1986; Abednego and Ogunlana, 2006; Awais et al., 2019). Effective and proper project management practices will help to gauge the feasibility and success rate of the CPEC. The budget and financing departments work coherently with the project management teams to find the important and effective means by which the project's success rate can be increased. These things support the economy of China and Pakistan. So, well-managed and financially secured project management initiatives will provide a strong base for the development and success of CPEC (Liu et al., 2021).

Relations will be developed internationally with CPEC success. Countries will coordinate with each other for trades. Foreign relations will also be strong due to trade. Trade level will be increased. New and equal opportunities will be created at the International Level (Chen, 2018; Farooq et al., 2018; Reed and Trubetskoy, 2019). Issues would be resolved on an urgent basis due to available facilities. Due to market competition vendor will improve the quality with the best price. It will provide a good environment for trade. Confidence upon each other's will be increased internally (Cheng et al., 2016). Countries relation will be stronger. The communication level will be increased internally. International relations are supported a lot by the CPEC and related initiatives. An example of this project's success is that in a mere period of 3 years, a new strategic partnership has invested a huge amount in this project. Saudi Arabia has joined this project as a third partner. This country is also interested in the development of the mining and natural resources industry, So, not only the business and industries are becoming popular and developed, but also a lot of sectors have improved due to the CPEC initiatives (Zimmerman, 2015; Sarfraz et al., 2018b; Zaman et al., 2021). The recent advancement in terms of highly skilled individuals' involvement in CPEC helped a lot to improve the confidence of Pakistani's. They are now easily communicating with foreign delegates (Chowdhary, 2015).

### Study Significance and Hypothesis Development

The combined efforts of Chinese and Pakistani workers showed a regional harmony to the world, the combined efforts in terms of the development of new and novel technologies have helped a lot in maintaining a good cooperative environment in between the people of both countries. The infrastructure development has helped a lot in reshaping the economy and related aspects of both countries' development (He et al., 2018). The exchange of skilled personals has created a lot of new social development initiatives. In recent studies, the conservation of resources and green engineering and technological aspects were discussed. It was observed that the exchange of social and economic resources had reshaped the overall economic condition of the whole Asian region. The other economies of the world have also invested in related projects of CPEC so it can be easily said that CPEC is not only hope and support for the Pakistani economy, but it is the gateway of industrial development and success for the whole world (Liu et al., 2016).

**Figure 1:**
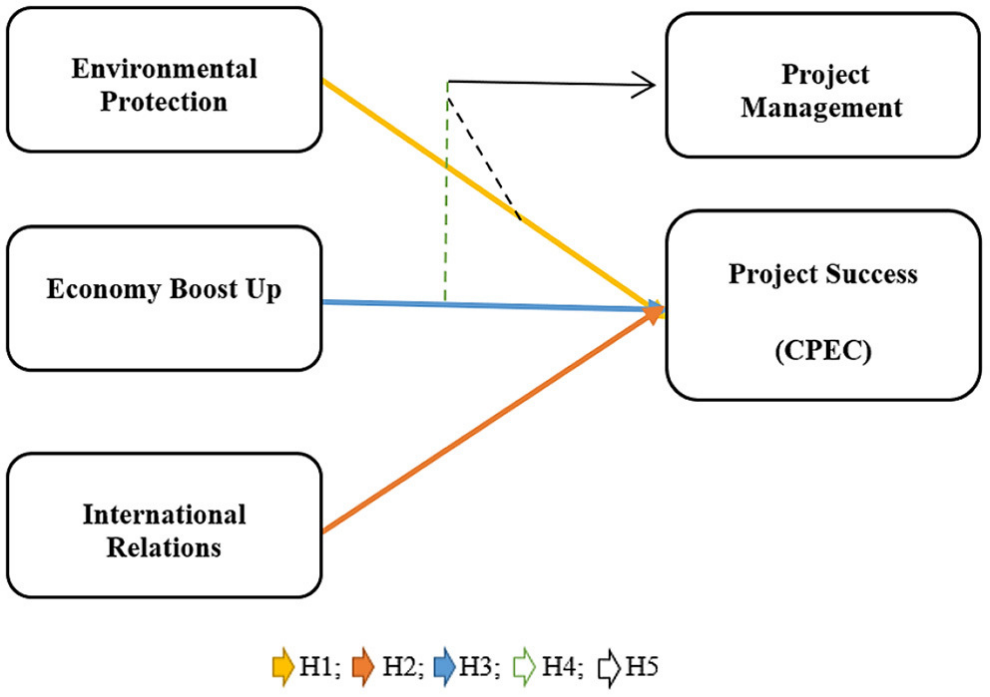
Hypothesis development and study framework.

Due to its geographical location, developing countries like Pakistan can serve as a tunnel for international bonding and development (Huo et al., 2021). The world needs such projects for enhancing the support and cooperation between different countries. Environmental protection, security and social harmony are some important advantages of this project. The hour's need is to indulge in all such projects with honesty, devotion, and hard work. Chinese and Pakistani governments are devoting a lot of efforts and time in this project. The timely audit and other regulatory strategies are necessary for the betterment of this huge project. If all the people work honestly, then CPEC will surely prove itself a new hope for the advancement of the economy of Pakistan and China (Kumar, 2007; Irshad, 2015; Ali et al., 2016; Nabi et al., 2017; Wang, 2017; Zubedi et al., 2018; Kakar and Khan, 2021). The hypotheses derived from the above studies are below here and can be seen in Figure 1.

H1: There is a positive association between environmental protection and overall success of CPEC project.

H2: There is a positive association between economy boost up and overall success of CPEC project.

H3: There is a positive association between international relations and overall success of CPEC project.

H4: Project management moderates the association between environmental protection and the overall success of the CPEC project.

H5: Project management moderates the association between economy boost up and overall success of CPEC project.

H6: Project management moderates the association between international relations and the overall success of the CPEC project.

### Methodology

CPEC is of great importance to both the countries (China and Pakistan) involved in this project. The quantitative methods have been used in this study to analyze data collected and examine the validity of the proposed hypotheses. Data has been collected from the site engineers, project managers, technical staff related to the CPEC projects. Moreover, the simple random sampling has been used to collect data, and the analysis of the data and the checking of validity of hypotheses, SMART-PLS, have been applied (smart-PLS, 2004). The required data has been acquired from the population of CPEC through the distribution of Questionnaires among them. A total of 550 questionnaires have been distributed through mail to collect data for our study while only 308 questionnaires have been returned within the 5 weeks on which our study is based on.

**Figure 2:**
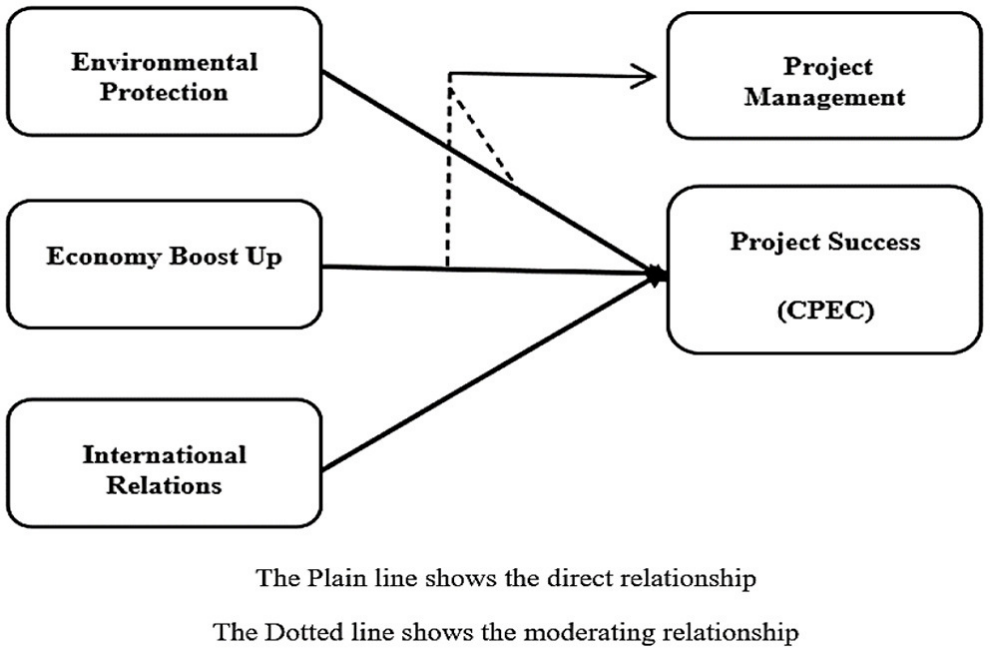
The study model.

This study addresses three indicators/factors like environmental protection, economy boost up, and international relations that consist of different items. The environment protection (EP) has six items (Kumar, 2007; Irshad, 2015), economy boost up (EBU) has three items (Brunner, 2013; Khetran and Saeed, 2017; Hao et al., 2020b), and the indicator international relations (IR) has four items (Rizvi, 2014; Chowdhary, 2015; Ali et al., 2020). In addition, the study addresses the project management (PM) as an important moderator between environment protection, economy boost up, and international relations and the success of CPEC project which consists of five items (Acemoglu, 2012; Nawaz et al., 2015; Menhas et al., 2019). The success of CPEC project (CPEC) is a dependent variable which has five items. These indicators are represented by the following Figure 2.

## Results

These findings provide the convergent validity for the assessment of the measurement model. The figures highlighted that high nexus between the items of the constructs and valid convergent validity because the loadings and AVE values are more than 0.50 while Alpha and CR values are also >0.70. These values are mentioned in Table 1.

**Table 1:** Convergent validity.

**Constructs**	**Items**	**Loadings**	**Alpha**	**CR**	**AVE**
Economy boost up	EBU1	0.848	0.775	0.821	0.606
	EBU2	0.760			
	EBU3	0.721			
Environmental protection	EP1	0.835	0.869	0.900	0.600
	EP2	0.731			
	EP3	0.727			
	EP4	0.785			
	EP5	0.763			
	EP6	0.802			
International relations	IR1	0.812	0.840	0.893	0.675
	IR2	0.849			
	IR3	0.821			
	IR4	0.804			
Project management	PM1	0.792	0.773	0.803	0.511
	PM2	0.513			
	PM4	0.747			
	PM5	0.772			
Success of CPEC	SCPEC2	0.782	0.827	0.898	0.747
	SCPEC4	0.910			
	SCPEC5	0.895			

The outcomes also provide the discriminant validity that is also a part of the measurement model, and it is checked by using Fornell-Larcker method and cross-loadings. The figures highlighted no high nexus between the variables and valid discriminant validity because the figures that exposed the nexus among variables are higher than those that show the nexus with other constructs. These values are mentioned in Tables 2 and 3.

**Table 2:** Fornell Larcker method.

	**EBU**	**EP**	**IR**	**PM**	**SCPEC**
EBU	0.778				
EP	0.471	0.775			
IR	0.559	0.628	0.822		
PM	0.482	0.548	0.554	0.715	
SCPEC	0.523	0.692	0.601	0.533	0.864

**Table 3:** Cross-loadings.

	**EBU**	**EP**	**IR**	**PM**	**SCPEC**
EBU1	**0.848**	0.442	0.554	0.471	0.481
EBU2	**0.760**	0.273	0.328	0.319	0.357
EBU3	**0.721**	0.368	0.394	0.314	0.369
EP1	0.393	**0.835**	0.508	0.548	0.652
EP2	0.408	**0.731**	0.578	0.414	0.595
EP3	0.397	**0.727**	0.371	0.443	0.567
EP4	0.329	**0.785**	0.491	0.354	0.446
EP5	0.338	**0.763**	0.482	0.348	0.400
EP6	0.290	**0.802**	0.476	0.375	0.466
IR1	0.330	0.505	**0.812**	0.452	0.515
IR2	0.365	0.554	**0.849**	0.441	0.534
IR3	0.558	0.489	**0.821**	0.477	0.455
IR4	0.617	0.511	**0.804**	0.456	0.463
PM1	0.389	0.402	0.417	**0.792**	0.404
PM2	0.263	0.282	0.308	**0.513**	0.270
PM4	0.363	0.480	0.482	**0.747**	0.465
PM5	0.349	0.365	0.343	**0.772**	0.345
SCPEC2	0.553	0.557	0.554	0.424	**0.782**
SCPEC4	0.409	0.627	0.496	0.485	**0.910**
SCPEC5	0.391	0.606	0.504	0.470	**0.895**

*EP, Environmental Protection; PM, Project Management; IR, International Relation; EBU, Economy Boost Up; SSPEC, Project Success (CPEC)*.

**Figure 3:**
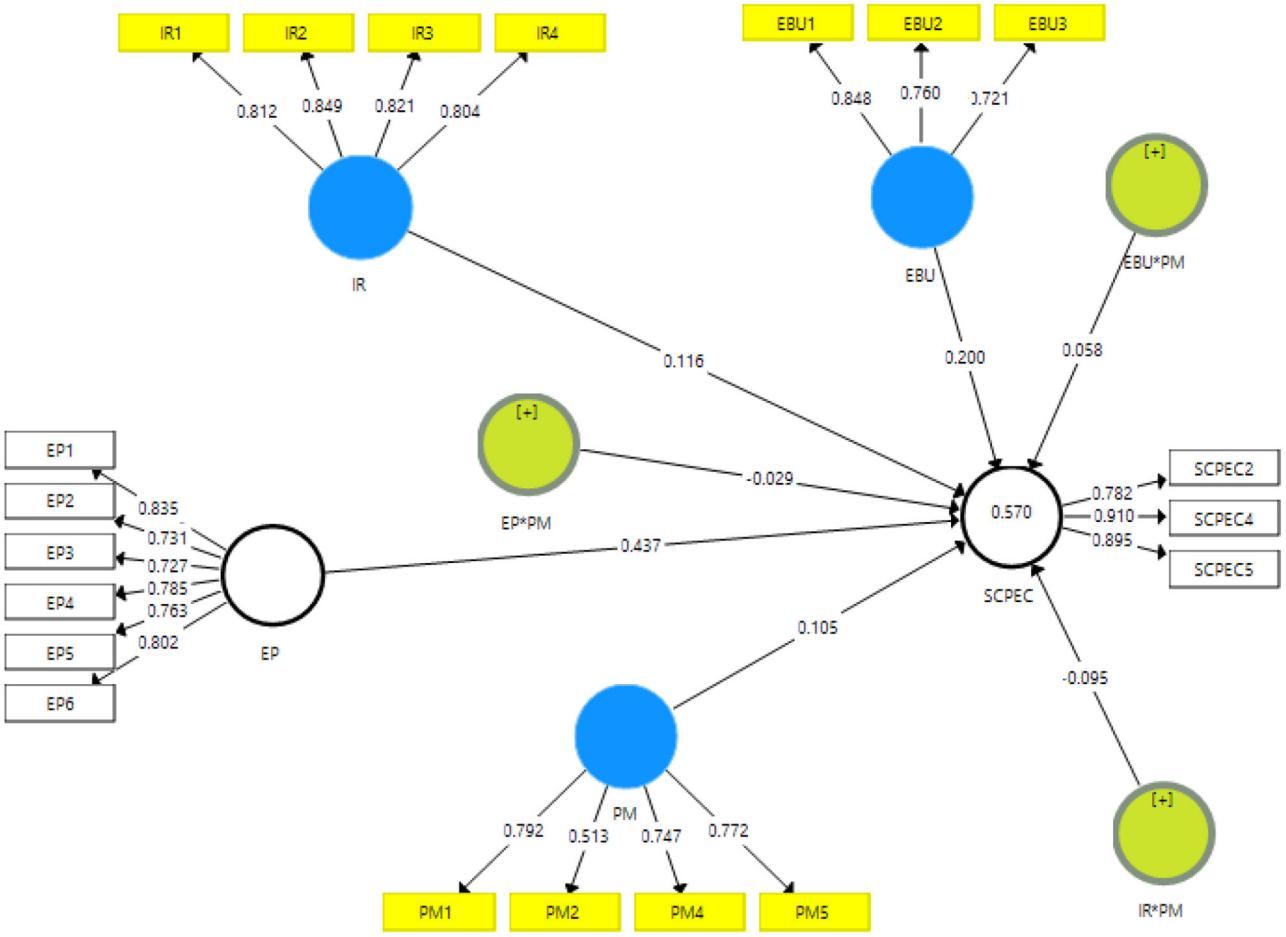
Measurement model assessment.

The discriminant validity is also checked by using Heterotrait-Monotrait (HTMT) ratio. The figures highlighted (see Figure 3) no high nexus between the variables and valid discriminant validity because the figures are less than 0.90. These values are mentioned in Table 4.

**Table 4:** Heterotrait Monotrait (HTMT) ratio.

	**EBU**	**EP**	**IR**	**PM**	**SCPEC**
EBU					
EP	0.593				
IR	0.737	0.728			
PM	0.697	0.684	0.727		
SCPEC	0.691	0.791	0.717	0.700	

The results revealed that environmental protection, economy boost up, international relations have a positive association with the success of CPEC and accept H1, H2, and H3. The outcomes also exposed that project management moderating among the nexus of economy boosts up, international relations, and success of CPEC and accepts H5 and H6. However, the outcomes also exposed that project management does not moderate among the nexus of environmental protection and success of CPEC and reject H4. These are highlighted in Table 5.

**Table 5:** Hypothesis results (Path Analysis).

**Relationships**	**Beta**	**S.D**.	**t-statistics**	***p*-values**	**L.L**.	**U.L**.
EBU -> SCPEC	0.200	0.037	5.368	0.000	0.120	0.266
EBU*PM -> SCPEC	0.058	0.032	1.811	0.043	0.004	0.124
EP -> SCPEC	0.437	0.033	13.256	0.000	0.380	0.504
EP*PM -> SCPEC	−0.029	0.033	0.885	0.378	−0.090	0.033
IR -> SCPEC	0.116	0.035	3.284	0.001	0.039	0.190
IR*PM -> SCPEC	−0.095	0.034	2.756	0.007	−0.164	−0.037
PM -> SCPEC	0.105	0.032	3.301	0.001	0.047	0.166

## Discussion and Implication

The results have indicated that environmental protection has positive impacts on the CPEC project. These results are in line with the previous studies (Akber, 2015; Nazir, 2015; Din, 2017), where it has been shown that the protection of the environment of both countries Pakistan and China has favorable impacts on the CPEC project. The results have shown that the economy boost up has a positive association with the CPEC project's success. These results match with the past studies of Nawaz et al. (2015), Ali et al. (2017), and Menhas et al. (2019), which has also shown that if the economic activities boost up the CPEC project is sound and successful.

The results have shown that international relations have a positive association with the success of the CPEC project. These results agree with past studies' results (Christoffersen, 2002; Khan et al., 2013; Rafi, 2016; Naz et al., 2018), according to which the sound relationship with foreign countries positively influences the CPEC project. The results have also represented that project management is a considerable moderator between international relations and the CPEC project's success. These results are in line with the past studies (Hao et al., 2020a,b), which shows that the project management's efficiency guarantees the CPEC project's success as it helps to protect the relations with other countries. The results have also revealed that the project management plays a moderating role between the economy boost up and international relations and CPEC management. These results match with the past studies of (Korytárová et al., 2015; Winter, 2016; Central, 2020).

The study carries both theoretical and empirical implications. The study contributes to the literature on international economic relations as it strengthens the economic relations between China and Pakistan in the form of the CPEC project's success. The study makes empirical implications as it guides both countries' governments to create successful their CPEC project. The study implies that Pakistan and China can make the CPEC project stronger with the efficient implementation of practices required to protect the environment, with the economic growth and boost up, and good strong relations with foreign countries.

## Concluding and Study Limitations

Environment protection has positive influences on the success of CPEC. The study examines that if both the countries involved in the CPEC project are protective, the project will be more successful. The results conclude that the economic boost-up is favorable for the CPEC project's success as the emerging economic activities and economic growth in both countries accelerate the trade between them. Moreover, the results prove that favorable relations with foreign countries positively affect the success of CPEC. The study also implies that project management is an appropriate moderator between environment protection, economic boost up, and international relations and the success of CPEC as it strengthens the mutual relationship between environmental protection, economic boost up, and international relations and the success of CPEC.

Though the study throws ample light on the CPEC project's success, it has several limitations. This study discusses only the three factors that influence CPEC projects' success, such as environment protection-related aspects, the economic boost up, and international relations. At the same time, many other factors affect the success of CPEC. Future scholars should expand the study's scope by addressing more factors in relation to the success of CPEC. Moreover, the study elaborates on the moderating influences of project management between environment protection, the economic boost up, and international relations and the success of CPEC. Future scholars should use project management as a mediator.

## Data Availability Statement

The original contributions presented in the study are included in the article/supplementary material, further inquiries can be directed to the corresponding authors.

## Author Contributions

TX, NG, and AN conceived and designed the concept and wrote the paper. SI and MA performed the literature review. AN and GA contributed in the data collection. JH and AM helped to provide technical support to collect the data. AN and TX contributed in analysis tools. TX has supervised the work. AN and AM reviewed the work to improve the outcomes. All authors have read and agreed to the published version of the manuscript.

## Conflict of Interest

The authors declare that the research was conducted in the absence of any commercial or financial relationships that could be construed as a potential conflict of interest.

## References

[B1] Abdullah M. I. Huang D. Sarfraz M. Sadiq M. W. (2021). Service innovation in human resource management during COVID-19: a study to enhance employee loyalty using intrinsic rewards. Front. Psychol. 12:247. 10.3389/fpsyg.2021.627659 33716893 PMC7947336

[B2] Abednego M. P. Ogunlana S. O. (2006). Good project governance for proper risk allocation in public–private partnerships in Indonesia. Int. J. Proj. Manag. 24, 622–634. 10.1016/j.ijproman.2006.07.010

[B3] Acemoglu D. (2012). Introduction to economic growth. J. Econ. Theory 147, 545–550. 10.1016/j.jet.2012.01.023

[B4] Akber A. L. I. (2015). China Pakistan economic corridor (CPEC): prospects and challenges for regional integration. Int. J. Soc. Sci. Humanity Stud. 7, 1–15. Available online at: https://dergipark.org.tr/en/pub/ijsshs/issue/26213/275986

[B5] Ali L. Mi J. Shah M. Shah S. J. BiBi K. (2017). The potential socio-economic impact of china Pakistan economic corridor. Asian Dev. Policy Rev. 5, 191–198. 10.18488/journal.107.2017.54.191.198

[B6] Ali L. Nawaz A. Iqbal S. Basheer M. A. Hameed J. Albasher G.. (2021). Dynamics of transit oriented development, role of greenhouse gases and urban environment: a study for management and policy. Sustainability 13, 1–14. 10.3390/su13052536

[B7] Ali W. Gang L. Raza M. (2016). China-Pakistan Economic corridor: current developments and future prospect for regional integration. Int. J. Res. 3. Available online at: https://papers.ssrn.com/sol3/papers.cfm?abstract_id=2794276

[B8] Ali Z. Gökçe Ö. Binark M. Gidreta A. D. (2020). China-Pakistan economic corridor and technicians of opinion in pakistani twittersphere: a thematic content analysis. Asian Stud. Int. J. Soc. Sci. 4, 9–28. Available online at: https://dergipark.org.tr/en/pub/asyar/issue/55675/744545

[B9] Awais M. Samin T. Gulzar M. A. Hwang J. (2019). The sustainable development of the china pakistan economic corridor: synergy among economic, social, and environmental sustainability. Sustainability 11:7044. 10.3390/su11247044

[B10] Brunner H.-P. (2013). What Is Economic Corridor Development and What Can It Achieve in Asia's Subregions Asian Development Bank Economics Working Paper Series, 117. Mandaluyong: Asian Development Bank.

[B11] Carvalho M. M. Rabechini R. Jr (2017). Can project sustainability management impact project success an empirical study applying a contingent approach. Int. J. Proj. Manag. 35, 1120–1132. 10.1016/j.ijproman.2017.02.018

[B12] Central G. (2020). Implication of the Developement of the Power Projects in Pakistan Under CPEC. Available online at: https://gwadarcentral.com/implications-of-the-development-of-power-projects-in-pakistan-under-cpec/ (accessed November 12, 2019).

[B13] Chakma B. (2019). The BRI and India's Neighbourhood. Strat. Anal. 43, 183–186. 10.1080/09700161.2019.1607030

[B14] Chaudhri M. A. (1986). Strategic and military dimensions in Pakistan-China relations. Pak. Horiz. 39, 15–28.

[B15] Chen X. (2018). Globalisation redux: can China's inside-out strategy catalyse economic development and integration across its Asian borderlands and beyond Cambridge J. Reg. Econ. Soc. 11, 35–58. 10.1093/cjres/rsy003

[B16] Chen Y. He L. Guan Y. Lu H. Li J. (2017). Life cycle assessment of greenhouse gas emissions and water-energy optimization for shale gas supply chain planning based on multi-level approach: case study in Barnett, Marcellus, Fayetteville, and Haynesville shales. Energy Convers. Manag. 134, 382–398. 10.1016/j.enconman.2016.12.019

[B17] Cheng X. He L. Lu H. Chen Y. Ren L. (2016). Optimal water resources management and system benefit for the Marcellus shale-gas reservoir in Pennsylvania and West Virginia. J. Hydrol. 540, 412–422. 10.1016/j.jhydrol.2016.06.041

[B18] Chowdhary M. (2015). China's billion-dollar gateway to the subcontinent: Pakistan may be opening a door it cannot close. Forbes. Usa, Forbes. Com.

[B19] Christoffersen G. (2002). The role of east asia in sino-american relations. Asian Surv. 42, 369–396. 10.1525/as.2002.42.3.369

[B20] Din A. N. U. (2017). Impact of emotional intelligence on project success with mediation of team cohesion and moderation of self-efficacy (Master Degree thesis). Capital University of Science and Technology, Islamabad, Pakistan.

[B21] Farooq M. S. Yuan T. Zhu J. Feroze N. (2018). Kenya and the 21st Century maritime silk road: implications for china-africa relations. China Q. Int. Strateg. Stud. 4, 401–418. 10.1142/S2377740018500136

[B22] Han C. Zhang B. Chen H. Wei Z. Liu Y. (2019). Spatially distributed crop model based on remote sensing. Agric. Water Manag. 218, 165–173. 10.1016/j.agwat.2019.03.035

[B23] Hao W. Mehmood S. Shah A. Nawaz A. Atif M. Noman S. M. (2020a). The impact of CPEC on infrastructure development, a-double mediating role of project success factors and project management. Argent. J. Psychol. Clin. 29, 737–750. 10.24205/03276716.2020.878

[B24] Hao W. Shah S. M. A. Nawaz A. Asad A. Iqbal S. Zahoor H.. (2020b). The impact of energy cooperation and the role of the one belt and road initiative in revolutionizing the geopolitics of energy among regional economic powers: an analysis of infrastructure development and project management. Complexity 2020:8820021. 10.1155/2020/8820021

[B25] He L. Shen J. Zhang Y. (2018). Ecological vulnerability assessment for ecological conservation and environmental management. J. Environ. Manag. 206, 1115–1125. 10.1016/j.jenvman.2017.11.059 30029345

[B26] Huo C. Hameed J. Nawaz A. Adnan Raheel Shah S. albahser G. Alqahtani W.. (2021). Scientific risk performance analysis and development of disaster management framework: a case study of developing Asian countries. J. King Saud Univ. Sci. 33:101348. 10.1016/j.jksus.2021.101348 PMC9042590 35495615

[B27] Initiative B. R. (2020). BRI Projects. Available online at: https://www.beltroad-initiative.com/projects/ (accessed October 28, 2020).

[B28] Irshad M. S. (2015). One belt and one road: dose China-Pakistan economic corridor benefit for Pakistan's economy J. Econ. Sustain. Dev. 6. Available online at: https://papers.ssrn.com/sol3/papers.cfm?abstract_id=2710352

[B29] Jia L.-C. Jin Y.-F. Ren J.-W. Zhao L.-H. Yan D.-X. Li Z.-M. (2021). Highly thermally conductive liquid metal-based composites with superior thermostability for thermal management. J. Mater. Chem. C. 9, 2904–2911. 10.1039/D0TC05493C

[B30] Kakar A. Khan A. N. (2021). The impacts of economic and environmental factors on sustainable mega project development: role of community satisfaction and social media. Environ. Sci. Pollut. Res. 28, 2753–2764. 10.1007/s11356-020-10661-y 32892282

[B31] Kanwal S. Pitafi A. H. Pitafi A. Nadeem M. A. Younis A. Chong R. (2019). China–Pakistan Economic Corridor (CPEC) development projects and entrepreneurial potential of locals. J. Public Aff. 19:e1954. 10.1002/pa.1954

[B32] Kanwal S. Rasheed M. I. Pitafi A. H. Pitafi A. Ren M. (2020). Road and transport infrastructure development and community support for tourism: the role of perceived benefits, and community satisfaction. Tour. Manag. 77:104014. 10.1016/j.tourman.2019.104014

[B33] Khan A. Waris M. Panigrahi S. Sajid M. R. Rana F. (2021). Improving the performance of public sector infrastructure projects: role of project governance and stakeholder management. J. Manag. Eng. 37:4020112. 10.1061/(ASCE)ME.1943-5479.0000886

[B34] Khan A. A. Ahmed M. Malik O. M. (2013). Pak-China economic alliance to bring prosperity in region. Int. Rev. Manag. Bus. Res. 2:776. Available online at: https://fac.ksu.edu.sa/sites/default/files/pak-china_economic_alliance_to_bring_prosperity_in_region.pdf

[B35] Khetran M. S. B. Saeed M. A. (2017). The CPEC and China-Pakistan relations: a case study on Balochistan. China Q. Int. Strateg. Stud. 3, 447–461. 10.1142/S2377740017500191

[B36] Khwaja M. A. Saeed S. Urooj M. (2018). Preliminary environmental impact assessment (EIA) study of China-Pakistan economic corridor (CPEC) northern route road construction activities in Khyber Pakhtunkhwa (KPK), Sustainable Development Policy Institute.

[B37] Korytárová J. Hanák T. Kozumplíková L. Špiroch M. (2015). Contribution of socio-economic benefits to economic efficiency of large-scale infrastructure projects, Proceedings of the 20th International Research Conference on Business, Economics and Social Sciences, IRC-2015 (Istanbul), 5–6.

[B38] Kumar S. (2007). The China–Pakistan strategic relationship: trade, investment, energy, and infrastructure. Strateg. Anal. 31, 757–790. 10.1080/09700160701662278

[B39] Li B.-H. Liu Y. Zhang A.-M. Wang W.-H. Wan S. (2020). A survey on blocking technology of entity resolution. J. Comput. Sci. Technol. 35, 769–793. 10.1007/s11390-020-0350-4

[B40] Li H. Hameed J. Khuhro R. A. Albasher G. Alqahtani W. Sadiq M. W.. (2021). The impact of the economic corridor on economic stability: a double mediating role of environmental sustainability and sustainable development under the exceptional circumstances of COVID-19. Front. Psychol. 11:634375. 10.3389/fpsyg.2020.634375 33569030 PMC7869893

[B41] Lin W. Chen B. Xie L. Pan H. (2015). Estimating energy consumption of transport modes in China using DEA. Sustainability 7, 4225–4239. 10.3390/su7044225

[B42] Liu S. Chan F. T. S. Ran W. (2016). Decision making for the selection of cloud vendor: an improved approach under group decision-making with integrated weights and objective/subjective attributes. Expert Syst. Appl. 55, 37–47. 10.1016/j.eswa.2016.01.059

[B43] Liu S. Yu W. Chan F. T. S. Niu B. (2021). A variable weight-based hybrid approach for multi-attribute group decision making under interval-valued intuitionistic fuzzy sets. Int. J. Intell. Syst. 36, 1015–1052. 10.1002/int.22329

[B44] Mamirkulova G. Mi J. Abbas J. Mahmood S. Mubeen R. Ziapour A. (2020). New Silk Road infrastructure opportunities in developing tourism environment for residents better quality of life. Glob. Ecol. Conserv. 24:e01194. 10.1016/j.gecco.2020.e01194

[B45] Maqsoom A. Babar Z. Shaheen I. Abid M. Kakar M. R. Mandokhail S. J.. (2021). Influence of construction risks on cost escalation of highway-related projects: exploring the moderating role of social sustainability requirements. Iran. J. Sci. Technol. Trans. Civ. Eng. 10.1007/s40996-021-00601-2. [Epub ahead of print].

[B46] Menhas R. Mahmood S. Tanchangya P. Safdar M. N. Hussain S. (2019). Sustainable development under belt and road initiative: a case study of China-Pakistan economic corridor's socio-economic impact on Pakistan. Sustainability 11:6143. 10.3390/su11216143

[B47] Nabi G. Khan S. Ahmad S. Khan A. Siddique R. (2017). China–Pakistan Economic Corridor (CPEC): an alarming threat to the biodiversity of Northern Pakistan. Biodivers. Conserv. 26, 3003–3004. 10.1007/s10531-017-1402-0

[B48] Nawaz A. Su X. Din Q. M. U. Khalid M. I. Bilal M. Shah S. A. R. (2020). Identification of the handamp;s (health and safety factors) involved in infrastructure projects in developing countries-a sequential mixed method approach of OLMT-project. Int. J. Environ. Res. Public Health 17:635. 10.3390/ijerph17020635 31963777 PMC7014267

[B49] Nawaz A. Su X. Nasir I. M. (2021). BIM Adoption and its impact on planning and scheduling influencing mega plan projects-(CPEC-) quantitative approach. Complexity 2021:8818296. 10.1155/2021/8818296

[B50] Nawaz A. Waqar A. Shah S. A. R. Sajid M. Khalid M. I. (2019). An innovative framework for risk management in construction projects in developing countries: evidence from Pakistan. Risks 7:24. 10.3390/risks7010024

[B51] Nawaz F. Azam M. F. Noor N. (2015). The dilemma of gadoon amazai industrial estate, khyber pakhtunkhwa. J. Econ. Sustain. Dev. 6, 313–327. Available online at: https://core.ac.uk/reader/234647040

[B52] Naz L. Ali A. Fatima A. (2018). International competitiveness and ex-ante treatment effects of CPEC on household welfare in Pakistan. Int. J. Dev. Issues. 17, 168–186. 10.1108/IJDI-05-2017-0100

[B53] Nazir M. (2015). Analysis of determinants for CPEC's success and failure: emerging challenges and lessons for Pakistan. Development 6, 200–207.

[B54] Rafi A. E. (2016). Completion of CPEC: Impact on Pakistan's Strategic Position and Economy. Islamabad: Islamabad Policy Research Institute.

[B55] Reed T. Trubetskoy A. (2019). Assessing the Value of Market Access from Belt and Road Projects. Policy Research Working Paper. Washington, DC: World Bank.

[B56] Rizvi H. A. (2014). The China-Pakistan economic corridor. Strateg. Stud. 34, 1–17. Available online at: https://www.issi.org.pk/wp-content/uploads/2015/12/Hasan-Askari-Rizvi_3435_SS_41_20142015.pdf

[B57] Sadiq M. W. Hameed J. Abdullah M. I. Noman S. M. (2020a). Service innovations in social media and blogging websites: enhancing customer's psychological engagement towards online environment friendly products. Rev. Argent. Clín. Psicol. 29, 677–696. 10.24205/03276716.2020.873

[B58] Sadiq W. Abdullah I. Aslam K. Zulfiqar S. (2020b). Engagement marketing: the innovative perspective to enhance the viewer's loyalty in social media and blogging e-commerce websites. Mark. Manag. Innov. 1, 149–166. 10.21272/mmi.2020.1-12

[B59] Sarfraz M. He B. Shah S. G. M. (2020a). Elucidating the effectiveness of cognitive CEO on corporate environmental performance: the mediating role of corporate innovation. Environ. Sci. Pollut. Res. 27, 45938–45948. 10.1007/s11356-020-10496-7 32808125

[B60] Sarfraz M. Ozturk I. Shah S. G. M. Maqbool A. (2020b). Contemplating the impact of the moderators agency cost and number of supervisors on corporate sustainability under the aegis of a cognitive CEO. Front. Psychol. 11:965. 10.3389/fpsyg.2020.00965 32536890 PMC7267056

[B61] Sarfraz M. Qun W. Abdullah M. I. Alvi A. T. (2018b). Employees' perception of Corporate Social Responsibility impact on employee outcomes: mediating role of organizational justice for Small and Medium Enterprises (SMEs). Sustainability (Switzerland) 10:2429. 10.3390/su10072429

[B62] Sarfraz M. Qun W. Hui L. Abdullah M. (2018a). Environmental risk management strategies and the moderating role of corporate social responsibility in project financing decisions. Sustainability 10:2771. 10.3390/su10082771

[B63] Sarfraz M. Shehzad K. Farid A. (2020c). Gauging the air quality of New York: a non-linear Nexus between COVID-19 and nitrogen dioxide emission. Air Qual. Atmos. Health 13, 1135–1145. 10.1007/s11869-020-00870-2 32837618 PMC7373842

[B64] Shahbaz M. S. Soomro M. A. Bhatti N. U. K. Soomro Z. Jamali M. Z. (2019). The impact of supply chain capabilities on logistic efficiency for the construction projects. Civ. Eng. J. 5, 1249–1256. 10.28991/cej-2019-03091329

[B65] Sial S. (2014). The China-Pakistan economic corridor: an assessment of potential threats and constraints. Confl. Peace Stud. 6:24. Available online at: https://www.researchgate.net/profile/Farhan-Zahid-2/publication/329759511_Understanding_the_Islamic_State_ideology_affiliates_and_the_Da%27esh_Model/links/5c19572892851c22a335c087/Understanding-the-Islamic-State-ideology-affiliates-and-the-Daesh-Model.pdf#page=12

[B66] smart-PLS (2004). Smart-PLS. https://www.smartpls.com/ (accessed December 02, 2020).

[B67] Ullah M. Khan M. W. A. Kuang L. C. Hussain A. Rana F. Khan A.. (2020). A structural model for the antecedents of sustainable project management in Pakistan. Sustainability 12:8013. 10.3390/su12198013

[B68] Wagner C. Tripathi S. (2018). India's Response to the Chinese Belt and Road Initiative: New Partners and New Formats. Berlin: Stiftung Wissenschaft und Politik.

[B69] Wang L. (2017). Opportunities and Challenges of the China-Pakistan Economic Corridor (CPEC) and Implications for US Policy and Pakistan. Washington, DC: East-West Center.

[B70] Winter T. (2016). One belt, one road, one heritage: cultural diplomacy and the Silk Road. The Diplomat 29, 1–5.

[B71] Wu C. Wu P. Wang J. Jiang R. Chen M. Wang X. (2021). Ontological knowledge base for concrete bridge rehabilitation project management. Autom. Constr. 121:103428. 10.1016/j.autcon.2020.103428

[B72] Wu H. Shah S. M. A. Nawaz A. Mahmood A. Albashar G. Shah S. A. R.. (2020). Disaster management policy development and engineering economics: an analysis of game-changing impact of COVID 19 on oil-power industry, environment, and economy. Rev. Argent. Clín. Psicol. 29:550. https://www.revistaclinicapsicologica.com/archivesarticle.phpid=162

[B73] Zaman U. Nawaz S. Anjam M. Anwar R. S. Siddique M. S. (2021). Human resource diversity management (HRDM) practices as a coping mechanism for xenophobia at transnational workplace: a case of a multi-billion-dollar economic corridor. Cogent Bus. Manag. 8:1883828. 10.1080/23311975.2021.1883828

[B74] Zeng S. X. Ma H. Y. Lin H. Zeng R. C. Tam V. W. Y. (2015). Social responsibility of major infrastructure projects in China. Int. J. Proj. Manag. 33, 537–548. 10.1016/j.ijproman.2014.07.007

[B75] Zimmerman T. (2015). The New Silk Roads: China, The US, and The Future of Central Asia. New York, NY: Center on International Cooperation.

[B76] Zubedi A. Jianqiu Z. Arain Q. A. Memon I. Khan S. Khan M. S.. (2018). Sustaining low-carbon emission development: an energy efficient transportation plan for CPEC. J. Inf. Process. Syst. 14, 322–345. 10.3745/JIPS.04.0067

